# Physiological, Chemical and Metabolite Profiling of *Pectobacterium carotovorum*-Inoculated Tomato Plants Grown in Nutrient-Amended Soils

**DOI:** 10.3390/plants14121876

**Published:** 2025-06-18

**Authors:** Sandra Maluleke, Udoka Vitus Ogugua, Njabulo Mdluli, Ntakadzeni Edwin Madala, Khayalethu Ntushelo

**Affiliations:** 1 Department of Agriculture and Animal Health, University of South Africa, Florida 1710, South Africa; 2 Department of Chemistry, University of South Africa, Florida 1710, South Africa; 3 Department of Biochemistry and Microbiology, University of Venda, Thohoyandou 0950, South Africa

**Keywords:** *Pectobacterium carotovorum*, tomato, nutrient assimilation analysis, photosynthesis, metabolomics, correlation analysis

## Abstract

This study evaluated the effects of a plant pathogenic bacterium *Pectobacterium carotovorum* strain BD163 inoculation and nutrient solution (CaCO_3_ (2 mM), NaCl (1 mM) and K_2_Cr_2_O_7_ (0.001 mM)) on the growth, photosynthesis, nutrient uptake and metabolomics of tomato seedlings. The experiment had four experimental treatments (1. solution + BD163 inoculation, 2. solution alone, 3. BD163 inoculation, 4. control). Plant growth and photosynthesis responses were minimal, and differences in nutrient assimilation and metabolite profiles were clear-cut. Of the photosynthesis parameters, only water use efficiency was impacted; it was higher in the bacterium-only treatment and unchanged in the other treatments. The quantities of boron, bismuth and nickel were affected, accumulating mostly in the “solution + BD163 inoculation” experimental set. Principal component analysis of metabolomics data separated the treatments into three groupings; group 1 was the double treatment, group 2 was the nutrient solution treatment and, finally, group 3 was the *P. carotovorum* and control treatments. Correlation analysis of the data showed an assumed interdependence of several plant factors. The authors concluded that the interaction between the bacterium, the plant and the nutrient solution is complex and more pronounced at the chemical and metabolite level than at the growth and photosynthesis level.

## 1. Introduction

Tomato (*Solanum lycopersicum* L.) is produced globally on a large scale and serves households, the fresh produce market and the processing industry. The crop is the second largest vegetable produced after potato. Recent accounts claim that tomato is produced on an estimated 5.9 million hectares of land and has an annual harvest of about 170 million tons [1]. The popularity of tomato results from its pleasant taste as part of various cooked dishes like stews, soups and relishes and as part of salads, sandwiches and sides in a variety of dishes. Tomato is highly nutritious, containing most essential nutrients, particularly provitamin A, folate, vitamin C, vitamin E, vitamin K and calcium [2]. On the downside, the tomato crop faces multiple physical, chemical and biological challenges that limit its production. Tomato may be exposed to toxicity and other varied soil conditions. These factors may influence its physiological processes, including its growth, photosynthesizing ability, nutrient assimilation and quantities of its metabolites [3]. Other factors impacting the tomato crop are pathogens. Pathogens infect the plant mostly during its vulnerable stages of growth. Upon entry into the plant, pathogens proliferate and cause symptoms, which cause tissue degeneration and plant death under severe circumstances. One of the known pathogens of tomato is *Pectobacterium carotovorum*, which, under favorable conditions of infection, induces soft rot disease [4]. *P. carotovorum* is classified as a Gram-negative bacterium within the taxonomic family Enterobacteriaceae, and the taxonomy of the Pectobacterium genus has been fine-tuned several times, for example, in the work performed by Gardan et al. [5]. Soft rot disease caused by *P. carotovorum* is characterized by a rapid accumulation of bacterial mucilage between cells, plant tissue breakdown and, finally, the soaking and oozing of the sap. Severe infections lead to plant collapse and death. Large-scale infection may cause epidemics, with devasting consequences of reduced crop productivity and food shortage. Despite the already vast knowledge about *P. carotovorum*, its effects on growth, photosynthesis, nutrient assimilation by the leaves and metabolite profiles in the tomato plant remain unknown. The picture is especially complex if plant inoculation with *P. carotovorum* is coupled with treatment with a mixture of select chemical compounds. Therefore, the purpose of this work was to assess the effect of *P. carotovorurm* and nutrient feeding on the growth, photosynthesis, nutrient assimilation and metabolite profiles of tomato plants. The study seeks to answer the following questions: What is the effect of *P. carotovorum* and a nutrient solution of CaCO_3_, NaCl and K_2_Cr_2_O_7_, applied singly and combined, on the growth of tomato seedlings? What is the effect of *P. carotovorum* and the nutrient solution of CaCO_3_, NaCl and K_2_Cr_2_O_7_ on the photosynthesis of tomato seedlings? What is the effect of *P. carotovorum* and the nutrient solution of CaCO_3_, NaCl and K_2_Cr_2_O_7_ on the metabolite profile of tomato seedlings? Which plant aspects of growth, photosynthesis and nutrient assimilation are similarly affected by the bacterial pathogen and nutrient solution treatments? We hypothesize that both the nutrient solution and the bacterium, either singly or in combination, affect the growth, photosynthesis, nutrient assimilation and metabolite profiles of tomato seedlings. Plants that received a nutrient solution of CaCO_3_, NaCl and K_2_Cr_2_O_7_ were also inoculated with *P. carotovorum*, alongside plants with either the nutrient solution only or the bacterium only and the control, which was treated with neither the nutrients nor the bacterium.

## 2. Results and Discussion

**Figure 1:**
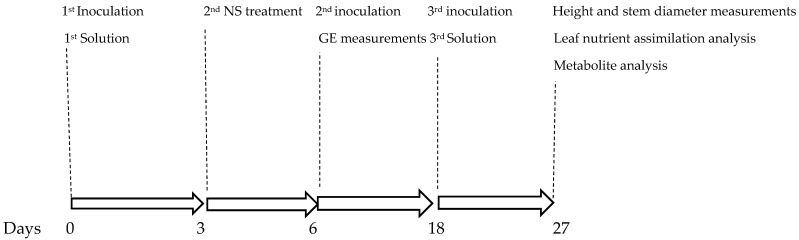
Timeline of the experiment. Tomato seedlings were inoculated with *Pectobacterium carotovorum* and were also treated with a mixture of elements and a nutrient solution (solution), which was CaCO_3_ (2 mM), NaCl (1 mM) and K_2_Cr_2_O_7_ (0.001 mM). Gas exchange (GE) measurements were taken on day 6, plant height and stem diameters were measured on day 27 and leaves were sampled on day 27.

**Figure 2:**
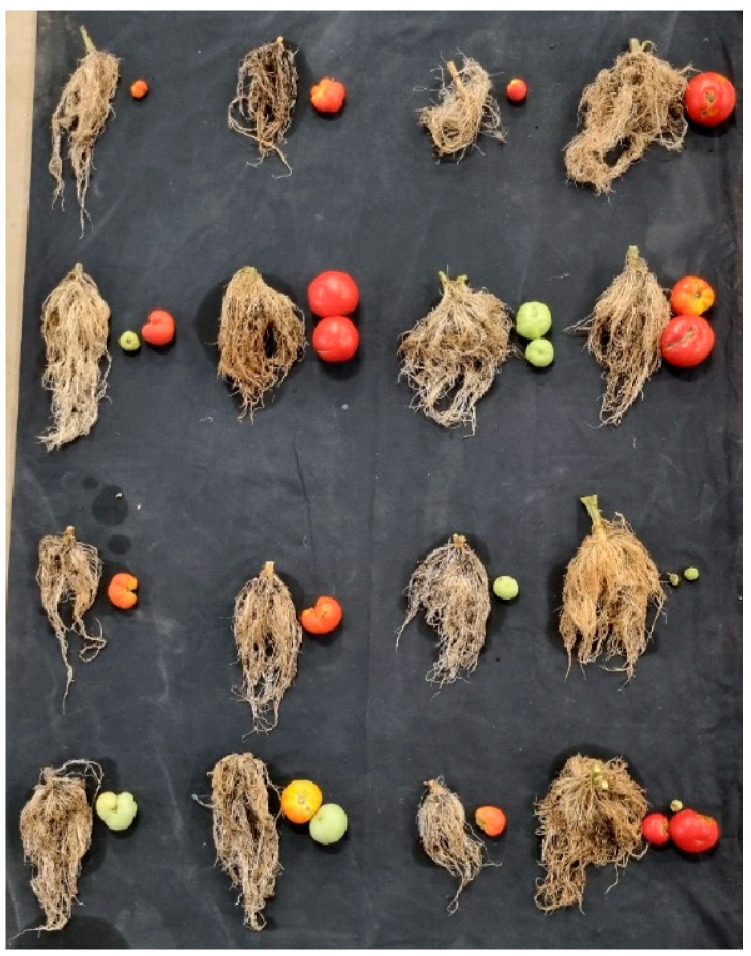
Roots and fruit of tomato inoculated with *Pectobacterium carotovorum* (strain BD163) and treated with a nutrient solution containing CaCO_3_ (2 mM), NaCl (1 mM) and K_2_Cr_2_O_7_ (0.001 mM) (column 1); only the nutrient solution (column 2); only BD163 (column 3); and the untreated control (column 4).

**Figure 3:**
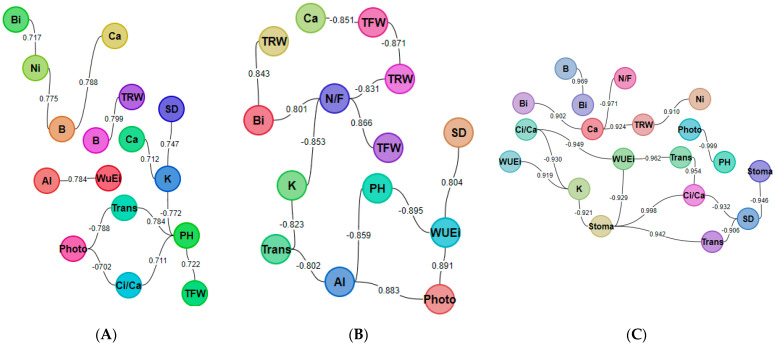
(**A**–**C**) Combined correlation network of the growth, gas exchange and elemental quantities of tomato seedlings from all experimental treatments, inoculated or not inoculated with *Pectobacterium carotovorum* (strain BD163) and treated or not treated with a nutrient solution containing CaCO_3_ (2 mM), NaCl (1 mM) and K_2_Cr_2_O_7_ (0.001 mM). (**A**) Correlation coefficients greater than or equal to 0.7 but smaller than 0.8 and correlation coefficients less than or equal to −0.7 but greater than −0.8. (**B**) Correlation coefficients greater than or equal to 0.8 but smaller than 0.9 and correlation coefficients less than or equal to −0.8 but greater than −0.9. (**C**) Correlation coefficients greater than or equal to 0.9 but smaller than or equal to 1.0 and correlation coefficients less than or equal to −0.9 but greater than or equal to −1.0.

**Figure 4:**
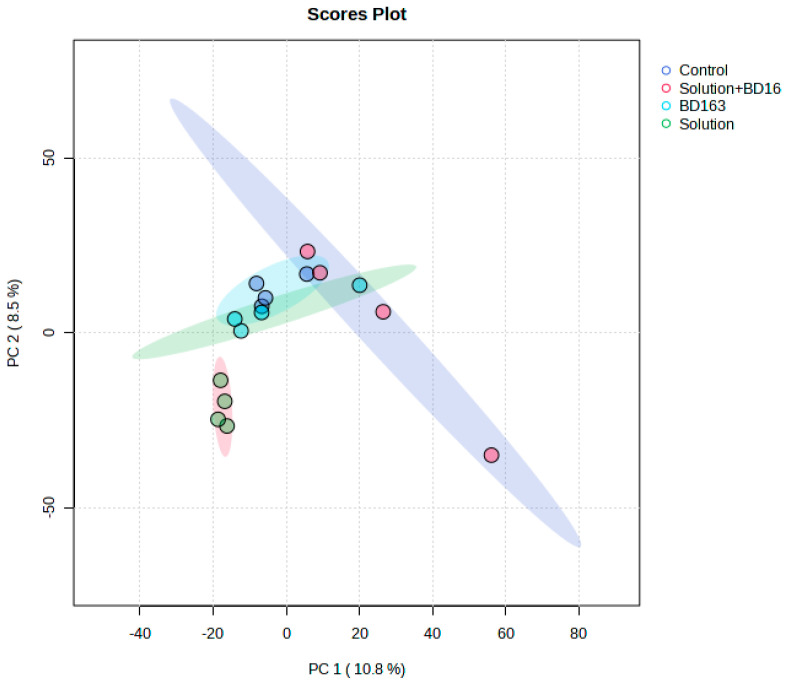
Computed principal component analysis (PCA) showing separated metabolic features of tomato leaves with four treatments, namely tomato inoculated with *Pectobacterium carotovorum* (strain BD163) and treated with a nutrient solution containing CaCO_3_ (2 mM), NaCl (1 mM) and K_2_Cr_2_O_7_ (0.001 mM) (solution + BD163—red circle), only the nutrient solution (solution—green circle) and only *Pc* (BD163—blue circle) and the untreated control (control—purple circle). Analysis with the LCMS-9030 qTOF was performed on leaves sampled 27 days after exposure to the treatments.

**Figure 5:**
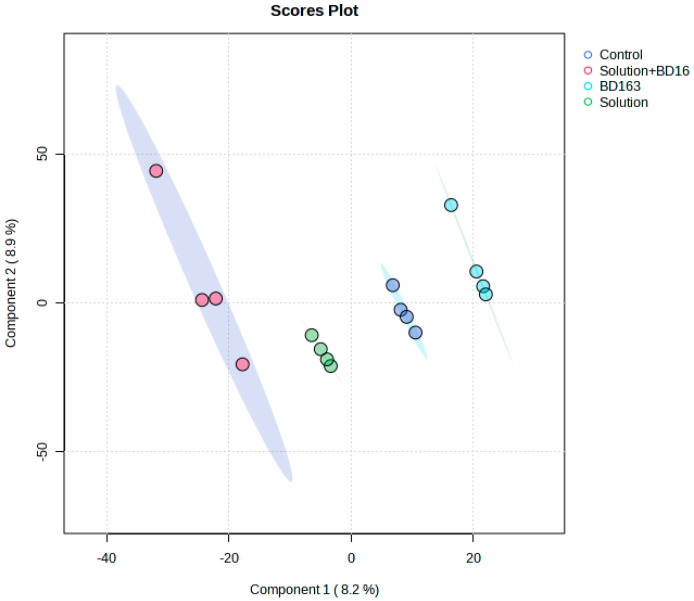
Computed partial least significant discriminant analysis (PLS-DA) showing separated metabolic features of tomato leaves with four treatments, namely tomato inoculated with *Pectobacterium carotovorum* (strain BD163) and treated with a nutrient solution containing CaCO_3_ (2 mM), NaCl (1 mM) and K_2_Cr_2_O_7_ (0.001 mM) (solution + BD163—red circle), only the nutrient solution (solution—green circle) and only *Pc* (BD163—blue circle) and the untreated control (control—purple circle). Analysis with the LCMS-9030 qTOF was performed on leaves sampled 27 days after exposure to the treatments.

**Figure 6:**
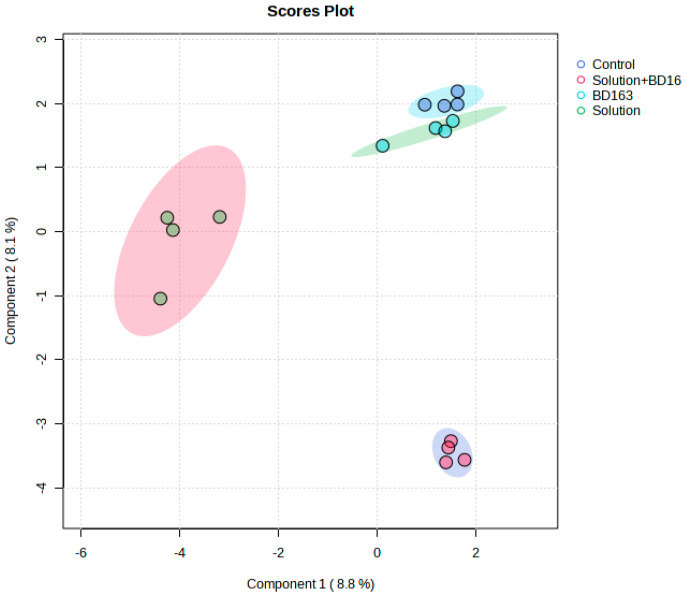
Computed sparse partial least significant discriminant analysis (SPLS-DA) showing separated metabolic features of tomato leaves with four treatments, namely tomato inoculated with *Pectobacterium carotovorum* (strain BD163) and treated with a nutrient solution containing CaCO_3_ (2 mM), NaCl (1 mM) and K_2_Cr_2_O_7_ (0.001 mM) (solution + BD163—red circle), only the nutrient solution (solution—green circle) and only *Pc* (BD163—blue circle) and the untreated control (control—purple circle). Analysis with the LCMS-9030 qTOF was performed on leaves sampled 27 days after exposure to the treatments.

**Figure 7:**
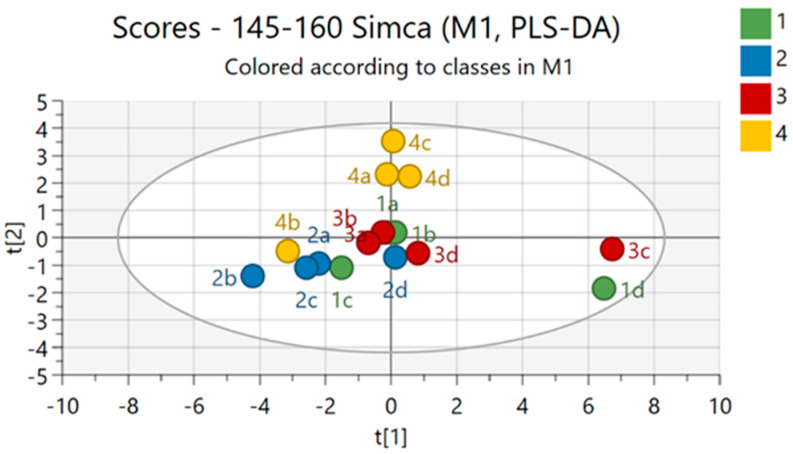
Nuclear magnetic resonance (NMR) spectroscopy data results. Computed partial least significant discriminant analysis (PLS-DA) showing separated metabolic features of tomato leaves with four treatments, namely tomato inoculated with *Pectobacterium carotovorum (Pc)* and treated with a nutrient solution containing CaCO_3_ (2 mM), NaCl (1 mM) and K_2_Cr_2_O_7_ (0.001 mM) (1—green square), only the nutrient solution (2—blue square) and only *Pc* (3—maroon square) and the untreated control (4—yellow square). Analysis with NMR spectroscopy was performed on leaves sampled 27 days after exposure to the treatments. The numbers and the accompanying letters are for the treatment and the plant. For each treatments plants were labelled a, b, c and d. X[1] = 0.602, R2X[2] = 0.16, Ellipse: Hotelling’s T2 (95%).

This experiment, conducted following the timeline illustrated in Figure 1, generated unique and novel results. The experimental treatments (1. nutrient solution + *Pectobacterium carotovorum* inoculation, 2. nutrient solution, 3. *P. carotovorum* inoculation, 4. control) caused responses that varied from minimal to distinct across the parameters measured (Figure 2 and Figure 3; Table 1, Table 2, Table 3 and Table 4). Except for water use efficiency (WUE), no effects on the growth of the plants or gas exchange parameters were recorded (Table 1 and Table 2). Administered alone, the bacterium increased WUE by to almost double that recorded in the rest of the treatments. As revealed by nutrient analysis, only the quantities of boron, bismuth and nickel were affected, out of the more than 20 elements assessed (Table 3). The quantity of boron was highest in the double treatment of the bacterium and the nutrient solution and lowest in the untreated set (control); bismuth followed a similar trend. Finally, nickel was higher in the double-treated plants than in the rest of the plants. Metabolite analysis performed with LCMS and NMR revealed underlying metabolite differences between the experimental treatments (Figure 4, Figure 5, Figure 6 and Figure 7, Supplementary Figures S1 and S2, Supplementary Table S1). Finally, correlation analysis of the measured parameters revealed various associations in the plants that received the unique treatments of *P. carotovorum*, the nutrient mixture and their combination (Figure 3 and Table 4).

### 2.1. Growth Measurements

The experimental treatments administered to the plants did not influence any of the plant growth parameters. This result cannot be understood as meaning that the bacterium and the nutrient solution do not affect growth. Factors other than the treatment may have determined this lack of response. Although seedlings were treated, it may be that the plants had passed the vulnerable stage in which a response to the treatment would occur. Degrees of susceptibility of plants to diseases at different growth stages is an established phenomenon, discovered in potato for purple top disease [6], in winter wheat and triticale for yellow rust [7], in wheat for the fungal pathogen *Cochliobolus sativus* [8] and in various other plant–pathogen associations. Given this background, we were therefore able to assume that our experimental tomato plants had grown past the vulnerable stage of growth. Moreover, pathogens that are expected to reduce plant growth are those that cause stunting as one of the symptoms, and Pectobacterium may be more of a soft rot pathogen than a stunting pathogen. A plant faces a dilemma when it is challenged with a pathogen; on the one hand, the plant must grow, and on the other hand, it must produce defense metabolites [9]. In situations where the plant prioritizes defense, it may suffer a growth penalty. However, *Ralstonia* wilt-resistant tomato plants were able to simultaneously promote growth and defense by switching processes that promote both processes [10]. *Pcc* is a soft-rotting bacterium, and therefore, its application on leaves was not expected to reduce growth especially if the plants had gone past the vulnerable stage. Secondly, it may be that the magnitude of the treatments was not sufficient to impact the plants. An association between inoculum levels and symptom expression is also an established phenomenon in plant–microbe interactions. A lack of response to the nutrient solution could also be caused by the application of a below-threshold concentration of the nutrient solution. It was unsurprising that the underlying plant processes, i.e., WUE, nutrient assimilation and metabolite responses, were affected by the treatments. Physiological and chemical processes are affected before symptom expression and effects on growth and yield.

### 2.2. Gas Exchange Responses

Of the measured gas exchange responses, photosynthetic rate, stomatal conductance, intercellular CO_2_ concentration, transpiration efficiency, Ci/Ca ratio and water use efficiency (WUE), only WUE, the amount of water lost to the amount of carbon fixed or the rate of net photosynthesis divided by the transpiration rate, was affected by the experimental treatments. The plants that were inoculated with *Pcc* had the highest, and significantly different, WUE of 208.79 ± 29.76a, whereas the WUE of the other treatments was between 114.73 ± 15.08 and 128.35 ± 15.08. *Pseudomonas syringae* pv. *glycinea* reduced the WUE of soybean with or without a bacterial consortium of 13 species collected from soybean leaves [11], virus infection of sugar beet is known to increase water use [12] and foliar disease generally reduces WUE [13]. In our study, the results were different from the results of the aforementioned studies in that the actions of *Pcc* increased WUE. A more comprehensive study on the effect of foliar pathogenesis on WUE in plants, including tomato, is needed. Such a study can answer the question as to under which circumstances a pathogen can increase or decrease WUE. It can be speculated that an increase in WUE is a coping mechanism by a plant after detecting that it is under pathogen attack. Why the bacterium could increase WUE in the presence of the nutrient solution is a matter for further investigation. However, it could be that the nutrient solution assisted the plant in canceling the effect of the pathogen, including plant signaling.

### 2.3. Nutrient Assimilation Analysis

The investigation has discovered changes in nutrient absorption caused by *P. carotovorum*. The observed changes in nutrient absorption in infected plants highlight the need to thoroughly understand these impacts to develop effective methods for protecting agricultural productivity from the adverse outcomes of bacterial infections. The observed changes in nutrient assimilation caused by *P. carotovorum* in inoculated tomato plants suggested that the bacterium disrupts the normal functioning of the plant’s nutrient acquisition. Decreased nutrient content and impaired photosynthetic activity could reduce plant growth and yield. It is plausible that the bacterium’s infection triggers physiological responses in the plant that divert resources away from nutrient assimilation processes. The experimental treatments affected the assimilation of boron and nickel. Boron accumulation was higher in the plants that were treated with just the nutrient solution. It was expected that boron would accumulate more in the leaves of the plants that received CaCO_3_ because CaCO_3_ improves the uptake of boron in plants [14]; however, the dynamics of boron uptake by a plant remain complex. With increased salinity, as was the case with the nutrient-solution-treated plants, the boron shoot concentration was elevated, as was the case in the study on broccoli presented in [15]. The uptake of nickel was higher in the plants that were treated with both the nutrient solution and the bacterium. Some bacteria influence the uptake of nickel by plants [16,17], and whether this is a repetition of the phenomena of the studies of Ma et al. [16] and Abou-Shanab et al. [17] cannot be confirmed. Hyperaccumulation of nickel in plants is important for plant defense against pathogens and herbivores [18]. Although nickel accumulation was significantly higher in the bacterial treatment with the nutrient solution, the authors of this publication could not establish any link between bacterial inoculation of plants and nickel accumulation, and therefore, the study of Ni and Punja [19] remains unconfirmed. The diminished effect of the pathogen *Pcc* on the inoculated tomato plants could be, in part, because of the uptake of boron and nickel since they are both known disease ameliorators [18,19]. However, this is just speculation and could not be established. The disease amelioration status of boron and nickel in tomato, with different pathogens under different conditions, may need further exploration with pathogenicity assays and omics experimentation. It was also noted that the uptake was higher in plants that received both the boron and the nutrient solution; the bacterium alone could not increase the uptake, nor could the solution alone increase it. Why both the bacterium and the nutrient solution had to be involved to increase boron accumulation remains surprising and requires a detailed study that varies the magnitudes of both treatments in various combinations. It could not be understood why the assimilation of the other elements was not affected. It could be due to either the treatments being lower than the threshold or the inability of the plant to absorb the nutrient solution for it to cause an effect. Twenty-four elements were selected for testing; however, the elements presented in Table 3 responded the most to the experimental treatment, although the quantities of some are not significantly different from the control. Not all the twenty-four elements chosen are present in the experimental nutrient solution. The research was not geared toward assessing the elements that were administered, but it was understood that the nutrient solution with its few elements can stimulate the assimilation of others.

### 2.4. Liquid Chromatography–Mass Spectrometry and Nuclear Magnetic Resonance Spectroscopy

Metabolite data generated with LCMS-9030 qTOF followed by principal component analysis (PCA), partial least squares discriminant analysis (PLS-DA), sparse PLS-DA and orthogonal PLS-DA showed differences between metabolite profiles of plants with the different experimental treatments. PCA was able to separate the treatments, but not as convincingly as the subsequent analysis with PLS-DA and OPLS-DA, which both showed clear chromatographic separation between treatments. Surprisingly, the bacterium treatment and the control had overlapping profiles in the PLS-DA, and the remaining treatments had distinct profiles. Pairwise comparisons performed using OPLS-DA showed clear chromatographic separation between each of the treatments and the control. Chromatographic separation of treatments by chromatography and mass spectrometry in tomato leaves challenged with a bacterial pathogen is not new, and the separation of plants that have received different treatment regimes is also not new. Our study agrees with a plethora of studies on the effect of bacterial pathogens on plants. A bacterial pathogen can induce metabolic changes in plants, as it was discovered by [20], using LC-MS and NMR, that *Pseudomonas syringae* induced metabolic changes in tomato with the rapid accumulation of various compounds, including phytoalexins and flavonoids. A similar study was performed by Lowe-Power et al. [21], who discovered induced metabolomic changes in tomato infected with *Ralstonia solanacearum.* Using untargeted GC analysis, Lowe-Power et al. [21] identified that 22 metabolites were enriched in sap of tomato plants inoculated with *R. solanacearum*. Using matrix-assisted laser desorption ionization–MS and LC-MS followed by multivariate analysis of the obtained the data, Galeano Garcia et al. [22] found tomato plants to respond to parasite infection by producing the crucial biomarkers tomatidine, saponins and isocoumarin with various response times from early to late infection. Reference [23] used NMR-based metabolomics profiling to discover virus- and bacteria-induced production of glycosylated gentisic acid, phenylpropanoids and a flavonoid (rutin). The first compound responded to viroid infection, whereas the latter two responded to bacterium infection. A multitude of other studies demonstrating the response of plants, particularly tomato, to infection are those presented in [24,25,26,27,28]. All these studies either use one or two applications e.g., LC-MS and/or GC-MS and/or NMR, and either perform an untargeted analysis or identify biomarkers that respond to infection. Some metabolites appear consistently as biomarkers of infection. An example of such a compound is rutin [29,30]. The primary purpose of our study was to assess plant response to *Pectobacterium* inoculation in the presence of a nutrient solution. The nutrient solution was crucial since nutrient assimilation was assessed to determine if bacterial inoculation influenced nutrient assimilation. Our experimental treatments are unique, and no study had ever been performed to assess the growth, photosynthesis and metabolite profiles of tomato treated with our special nutrient solution and *Pcc*. Our results on the metabolomic response to the pathogen are unsurprising and firmly agree with results from previous studies. The response of the plant to the nutrient solution uncovered a new avenue in plant nutrition responses. It revealed that boron accumulated more when the plant was treated with the nutrient solution and with the bacterium and the nutrient solution. Nickel accumulation was greater when both the bacterium and the nutrient solution were present. The chromatographic separation in the LC-MS and NMR analysis could then be attributed mostly to boron and nickel accumulation. With regard to nutrition and metabolic responses, notable studies are those by Sung [31,32] and Iglesias [33]. The factors investigated in the present study attempted to uncover the complexity of plant response to the treatments of *Pectobacterium* and nutrients. One of the interesting aspects was the discovery of kaempferol-3-O-rutinoside, a related structural analog of rutin, as a downregulated compound. Rutin has been featured in various studies as a disease-response compound. López-Gresa [23] discovered a rapid accumulation of the flavonoid rutin after infiltration of tomato plants with *Pseudomonas syringae*. Rutin was identified in methanolic extracts of leaves, stems and roots of four tomato cultivars exhibiting a spectrum of intermediate to high resistance to *R. solanacearum* [34]. Tomato infected with *Botrytis cinerea* showed higher concentrations of flavonoids, such as rutin and quercetin-3-galactoside. Similarly, rutin was identified in potato leaves resistant to late blight. In addition, an NMR-based study showed that rutin was the flavonoid that was most expressed in response to *P. syringae* infection in tomato, and in potato plants, it is associated with late blight resistance of different cultivars [23,35,36,37]. Similar other studies are those conducted by Lee et al. [24] and Sade et al. [26].

The quantity of flavonoids in a plant can change after exposure to stress and photoperiods [38,39], among others. In pepper plants exposed to chilling, Wang et al. [38] found that, generally, the contents of total phenols and flavonoids initially increased during the cold storage period and later declined. The discovery of Wang et al. [38] means that the plight of the flavonoid of interest for disease response could not be understood by assessing secondary metabolites only at experimental termination on day 27 after initial inoculation, as was the case with this study. Flavonoids are a versatile class of bioactive compounds [40] and are involved in non-enzymatic defense against plant stress [41], and thus, it is important to assess their quantifies in a study where the response of the plant to stress is gauged. Assessing the quantities of metabolites once is a drawback of the current study, and this must be considered in designing future studies. Despite this limitation, this study has revealed differences in the metabolomic profiles of the four different experimental treatments.

### 2.5. Correlation Analysis of Measured Variables

Some of the strong correlations were expected, and others were not (Table 4, Figure 3). Total fruit weight and the number of fruits were positively correlated (0.866); total root weight was negatively highly correlated with the number of fruits per plant (−0.831) and total fruit weight (−0.871). The rate of photosynthesis was negatively correlated with plant height (−0.999), and stomatal conductance was negatively correlated with stem diameter (−0.946). The transpiration rate was negatively correlated with stem diameter (−0.906) but positively correlated with stomatal conductance (0.942). Ci/Ca was negatively correlated with stem diameter (−0.932) but positively correlated with both stomatal conductance (0.998) and transpiration rate (0.954). Of all the parameters measured and presented on the correlation table, water use efficiency (WUE) correlated highly with most plant functions, namely plant height (−0.895), stem diameter (0.804), photosynthesis rate (0.891), transpiration rate (−0.962), stomatal conductance (−0.929) and Ci/Ca (−0.949). Aluminum concentration was highly correlated with plant height (−0.859), the rate of photosynthesis (0.883) and transpiration rate (−0.802). Bi was highly correlated with the number of fruits (−0.801), total root weight (0.843) and B (0.969). Surprisingly, the leaf quantity of Ca was negatively highly correlated with the number of fruits (−0.971) and total fruit weight (−0.851), and unsurprisingly, it was positively correlated with total root weight (0.924) and Bi (0.902). Potassium on the other hand was highly correlated with the number of fruits per plant (−0.853), stomatal conductance (−0.921), transpiration rate (−0.823), Ci/Ca (−0.930) and WUE (0.919). Finally, Ni was positively correlated with total root weight (0.91). The correlation coefficients are presented in Table 4 and Figure 3. In a study by Hacisalihoglu et al. [42], inoculation of tomato plants with *Ralstonia solanacearum* reduced the accumulation of calcium and boron in tomato leaves. Moreover, a study [43] showed that foliar sprays of calcium boosted tomato yield parameters compared to the unsprayed control, and this meant that calcium is linked to yield boosting in tomato. Similarly, Sajid et al. [44] also found that calcium in tomato leaves boosts the growth, yield and quality of tomato. It also improves leaf and root vigor [28], and therefore, it is unsurprising that there is a high positive correlation coefficient between the calcium concentration of the plant and root dry weight, and the high negative correlations between the Ca concentration and the total fresh weight and number of fruits per plant remain surprising. Potassium was found to boost yield in a study investigating the effect of potassium/calcium balance on tomato yield [45], which is why it is was correlated with total fruit weight and total fruit number in this study. In the present study, the potassium quantity of the leaves correlated negatively with the number of fruits per plant (−0.853), stomatal conductance (0.921), transpiration rate (−0.823) and Ci/Ca (−0.930). Unsurprisingly, potassium correlated strongly and positively with WUE (0.919). Potassium has a complex role in the plant; it is a macronutrient representing up to 10% of the dry weight of the plant [46] and a major inorganic cation in the cytoplasm, responsible for the activity of various enzymes, some of them involved in primary metabolism reactions [47], and it is involved in solute transport, stomatal functioning [30,31], cell integrity and cell-to-cell maintenance [48,49,50,51]. Pathogen infection of a plant can disturb the intricate equilibrium necessary for healthy plant growth by exerting an influence on the uptake of nutrients. In general, it is unsurprising that many high correlations between the measured parameters of growth, photosynthesis and nutrient assimilation were discovered. A plant is a physical continuum, and therefore changes in one aspect of a plant can cause changes in others. Growth depends on photosynthesis; photosynthesis depends on enzyme function, which is controlled by coenzymes that depend on nutrient uptake. The nutrient mixture was selected to combine numerous elements detectable by ICP OES. In addition, various combinations of CaCO_3_ and micronutrients are effective against tomato wilt caused by *Ralstonia solanacearum* [52]. Pot-planted tomato plants survive on 20% w/w of CaCO_3_/soil [53], and therefore, 2 mM CaCO_3_ was still within the non-toxic range. A recommended dose of CaCO_3_ for pot-grown tomato is not easily obtainable, and therefore, the authors of the current manuscript opted for a non-toxic nutrient solution treatment and monitored the plants after the initial solution application. In addition to the general benefits of NaCl for tomato plants, NaCl also boosts the organoleptic properties of tomato fruit [54], and tomato plants survive in salinity levels as high as 121 mM Na^+^ and 120 mM Cl^−^ [55], which therefore permitted us to administer 1 mM NaCl around the stem base of tomato plants. Varying concentrations of K_2_Cr_2_O_7_ (0.05, 0.5, 1, 5, 10 mgL^−1^ of irrigation water) had varied morpho-physiological responses in the tomato plant, with all treatment plants surviving the highest concentrations [56]. Based on its effect on the morpho-physiological aspects of tomato, K_2_Cr_2_O_7_ was another informed choice for inclusion in our nutrient mix, and our choice of 0.001 mM posed no danger to the plants. However, formulating a nutrient mix for the current study remained a challenge and warrants further investigation where the components of the mixture are decoupled and a new formulation with various concentrations of every compound is included. The compound rutin or phytomelin is consistently detected in studies of the response of tomato plants to infection. In the present study, rutin is downregulated, and the authors of the present manuscript feel rutin needs to be studied in isolation to understand its role in plant–pathogen studies (Supplementary Table S1). 2-Hydroxyhexadecanoic acid (Supplementary Table S1) could be produced by the bacterium itself, as the study of Varbanets et al. [57] showed it to be produced by another plant pathogen, the bacterium *Ralstonia solanacearum*. Quinic acid, another metabolite detected (Supplementary Table S1), is linked to plant defense in various studies, notably the study by Lui et al. [58], which discovered that quinic acid induces hypovirulence and expression of a hypovirulence-associated double-stranded RNA in *Rhizoctonia solani* after testing on potato. These three metabolites, namely rutin, 2-Hydroxyhexadecanoic acid and quinic acid, should form part of future studies to elucidate their role in tomato plants inoculated with a bacterium in the presence or absence of a nutrient solution. It is unclear how Pectobacterium influences nutrient uptake; however, bacterial growth is characterized by slime in the intercellular spaces and in the vascular tissues of the plant. Bacterial colonization of the vascular tissue blocks the nutrient pathway, and therefore the leaves are expected to be starved of nutrients. The situation with the current study may not be the same since the bacterium was pressure-infiltrated through the leaves and may not have sufficient mass to block the nutrient pathway. The differential assimilation of nutrients observed could have been a result of signaling before the bacterium built its mass in the vascular tissue. This can only be speculated until a follow-up investigation is conducted. The role of the nutrient solution administered to the plant could also be speculated in the vast arena of the plant system. This study does not have much agricultural application since it was intended to investigate phenomena at a physiological and biochemical level. The nutrient solution was not administered to investigate its potential to ward off the effects of the bacterial pathogen. Given the treatments administered in the present study, it can be concluded that the interaction between the bacterium, the plant and the nutrient solution is complex and more pronounced at the chemical and metabolite level than at the growth and photosynthesis level. Recall that plant growth and photosynthesis responses were minimal, and differences in nutrient assimilation and metabolite profiles were clear-cut. Of the photosynthesis parameters, only WUE was impacted; it was higher in the bacterium-only treatment and unchanged in the other treatments. The quantities of boron, bismuth and nickel were affected; however, they accumulated mostly in the “solution + BD163 inoculation” experimental set. Principal component analysis of metabolomics data separated the treatments into three groupings, group 1 was the double treatment, group 2 was the nutrient solution treatment and, finally, group 3 was the *P. carotovorum* and control treatments. Correlation analysis of the data showed an assumed interdependence of several plant factors. This was evident given the high number of correlation coefficients either less than −0.7 or greater than 0.7.

## 3. Materials and Methods

### 3.1. Plant Material

Sixteen tomato seedlings of the commonly grown cultivar Heinz 1370, raised in an organically certified potting soil mix, were selected for study. The plants were maintained in a glasshouse at temperatures of approximately 25 °C day and 20 °C night, regulated using the wall-wetting function of the Priva control system (Priva Intégro v. 730, Priva, De Lier, The Netherlands). Lighting followed natural day/night light/darkness regimes, and water for irrigation was applied manually around the stem base.

### 3.2. Experimental Treatments

The 16 plants were divided into four groups. The first group was inoculated with the bacterium, *Pectobacterium carotovorum* (strain BD163), coupled with treatments of a nutrient solution containing CaCO_3_ (2 mM), NaCl (1 mM) and K_2_Cr_2_O_7_ (0.001 mM) dissolved in distilled water. The second group of four plants was treated with only the nutrient mixture without *P. carotovorum*. The third group was treated with just the bacterium with no nutrient mixture. The final group received neither the bacterium nor the nutrient mixture. The experimental treatments were distinguished as 1. solution + BD163, 2. solution 3. BD163 and 4. control. The bacterium and the nutrient solution were administered three times. Following the first treatment, subsequent treatments were ad hoc, after a visual observation of whether the plant was reacting to the treatment or not. When it was observed that the plants were not responding to treatment, the second application was performed 6 days after the first, and the third 12 days after the second (Figure 1). Throughout the experiment, until the trial was terminated and roots and fruits were assessed and photographed (Figure 2), the plants were continuously randomized to counter non-uniform conditions in the glasshouse.

### 3.3. Bacterial Inoculation and Nutrient Mixture Application

The bacterial inoculum was prepared from an overnight Luria Bertani broth culture. The liquid culture was centrifuged, the pellet was recovered and resuspended in buffer (0.0014 M KH_2_PO_4_, 0.0025 M Na_2_HPO_4_, pH 7.00) and the cell count was adjusted, with the inoculation buffer, to 10^8^ CFU. Plants were inoculated by pressure infiltration on the underside of the leaf. The uninoculated experimental sets were mock-inoculated with just the buffer. Lower leaves of uniform appearance were selected for inoculation, and pressure infiltration was performed with a hand syringe until the bacterial culture permeated beneath the epidermis. The infiltration was usually performed for a few seconds. The nutrient mixture (CaCO_3_ (2 mM), NaCl (1 mM) and K_2_Cr_2_O_7_ (0.001 mM) dissolved in distilled water) was administered on the base of the stem, 10 mL on the first two applications and 5 mL on the last application. Irrigation was skipped during the days of nutrient feeding, and experimental sets not earmarked for the nutrient mixture received an equal amount of distilled water.

### 3.4. Gas Exchange Measurements and Nutrient Assimilation Determination

Fully expanded leaves showing robust growth were selected for gas exchange measurements. The gas exchange measurements taken were photosynthesis rate, stomatal conductance, intercellular CO_2_ concentration, transpiration efficiency, Ci/Ca ratio and water use efficiency. Two leaves were read for each plant, and a portable infrared gas analyzer, the LI-COR Photosynthesis System (Li-6400, Li-Cor Inc., Lincoln, NE, USA) was used to capture the readings. For nutrient assimilation analysis, leaf samples quenched in liquid nitrogen were ground, and a total of 100 mg of the sample was treated with nitric acid. Digestion was facilitated using the Anton Paar Multiwave 5000 (Perkin Elmer, Whatman, MA, USA) at 180 °C for an hour. After microwave treatment, the samples were allowed to cool before undergoing analysis using inductively coupled plasma optical emission spectroscopy (PerkinElmer) to determine the quantities of 24 elements including, aluminum, boron, bismuth, calcium, potassium and nickel, following the procedure outlined by Nnabuo-Eguzozie et al. [59].

#### Data Analysis

Growth data, photosynthesis data and nutrient assimilation data were analyzed using analysis of variance followed by the Duncan multiple range test to distinguish between treatments at (*p* < 0.05). Data was analyzed using STATISTICA software 10 and is presented in Table 1, Table 2 and Table 3. The correlation of measured variables was assessed and is presented in Table 4, and a nodal network was generated to show all correlations ≥ 0.7 and ≤−0.7 (Figure 3). Both nutrient assimilation analysis in leaf material and secondary metabolite profiling were conducted 27 days after the first round of experimental treatments.

### 3.5. Liquid Chromatography

Liquid chromatography–quadrupole time-of-flight tandem mass spectrometry (LCMS-9030 qTOF (Shimadzu Corporation, Kyoto, Japan)) was used to analyze leaves sampled 27 days after the tomato plants were first inoculated and treated with the nutrient solution. The extraction of secondary metabolites was performed using methanol, and chromatographic separation at 55 °C was conducted using a Shim-pack Velox C18 column with dimensions of 100 mm × 2.1 mm and a particle size of 2.7 µm (Shimadzu Corporation, Kyoto, Japan). The analyte injection volume was 5 µL, and analysis was performed following a binary mobile phase (solvent A (0.1% formic acid in Milli-Q HPLC-grade water) (Merck, Darmstadt, Germany) and solvent B (UHPLC-grade methanol with 0.1% formic acid) (Romil Ltd., Cambridge, UK)). Elution was performed with 10% solvent B for 3 min, followed closely by a gradual increase in solvent B to reach 60% over three minutes. The proportion of solvent B was further increased to 90% over 3 min and maintained at this level for an additional minute. For system restoration to its initial state, the conditions were then adjusted to 60% B within 1 min and held constant for an additional minute. This procedure facilitated the re-establishment of equilibrium within the column. Chromatography was performed with a qTOF high-definition mass spectrometer configured to utilize negative electrospray ionization. The settings of the mass spectrometer were as follows: nebulization, interface voltage (3 kV), interface temperature (300 °C), dry gas flow (0.45 L/min), detector voltage (1.8 kV), heat block (400 °C), DL (280 °C) and flight tube (42 °C) temperature. Ions were fragmented using argon as the collision medium, following the methodology adopted by Ramabulana et al. [60]. The mass spectrometry data obtained from the Shimadzu LCMS-9030 qTOF was pre-processed using a cloud-based bioinformatics program, XCMS Online, employing HPLC/UHD-qTOF parameters. The centWave feature detection method was used, with a maximum threshold of 15 ppm and a signal-to-noise ratio of 6. Prefiltering was conducted based on intensity and noise levels, with thresholds of 100 and 3, respectively. Obiwarp was employed for correcting retention time in conjunction with profStep. The alignment process utilized a minimum proportion of samples of 0.5 and a width of 0.015 *m*/*z*. The data was analyzed using the Kruskal–Wallis statistical test, which generated a Microsoft Excel table of the obtained results. The resultant table was transposed and saved as a comma-delimited (CSV) file and uploaded to the internet-based platform MetaboAnalyst v5.0; the data was log-transformed, Pareto-scaled and analyzed to generate principal component analysis (PCA), partial least squares discriminant analysis (PLS-DA), sparse PLS-DA and orthogonal PLS-DA plots (Figure 4, Figure 5 and Figure 6, Supplementary Figures S3–S5); the metabolites were further annotated, and the quantities were correlated (Supplementary Figures S1 and S2; Supplementary Table S1).

### 3.6. Secondary Metabolites Identification and Quantification

To annotate metabolic peaks after separation and detection on the LCMS-9030 qTOF, the raw mass spectrum data was analyzed using SIRIUS Software, version 5.5.7. The OPLS-DA S-plots were used to select the most important metabolites that needed to be annotated. The S-plot provides a graphical depiction of the covariance and correlation seen in the OPLS-DA scatter plot. Therefore, the lists of upregulated and downregulated compounds that were found to be statistically significant and reliable were derived from the S-plots. These compounds were then annotated putatively in accordance with level 2 of the Metabolomic Standards Initiative (MSI) [61]. Using the SIRIUS identification tool [62], the retention times and exact masses of the samples were compared to those of the reference compounds, with maximum deviations allowed at 0.1 min and 10 ppm. The compound composition of the samples was searched and verified in various online libraries/databases. The annotation was based on the interpretation of mass fragmentation patterns, MS/MS spectra, mass spectral library searches and the published literature and datasets, as previously performed [63]. Furthermore, annotation was conducted by analyzing mass fragmentation patterns and MS/MS spectra, carrying out searches in mass spectral libraries, and referring to the existing literature and datasets [63]. The quantity of the annotated metabolites from both untreated and treated plants was adjusted to the total ion count of each sample and then converted using a logarithm of base 2. The fold change was then determined. The results of this analysis appear in Supplementary Figures S1 and S2 and Supplementary Table S1.

### 3.7. Nuclear Magnetic Resonance Spectroscopy

Leaves sampled 27 days after the tomato plants were first inoculated and treated with the nutrient solution were crushed in liquid nitrogen using a mortar and pestle. Deuterated solvents were used to extract secondary metabolites, which were analyzed using an 800 MHz Varian NMR spectrophotometer. The NMR spectrophotometer produced ^1^H intensity spectra that were analyzed further. Mestrenova, version 15.0.1-35756 was used to perform preliminary processing on NMR spectra, allowing the extraction of signal intensity values. Of the chemometric procedures performed using MetaboAnalyst 5.0, PLS-DA categorized the samples based on the NMR intensity values and successfully discriminated the samples based on the experimental treatments (Figure 7).

## Figures and Tables

**Table 1:** Measurement of growth and yield parameters of tomato plants inoculated with *Pectobacterium carotovorum* (BD163) and treated with a nutrient solution containing CaCO_3_ (2 mM), NaCl (1 mM) and K_2_Cr_2_O_7_ (0.001 mM) (solution). Letters accompanying the measurements indicate statistically significant differences at (*p* < 0.05) *n* = 4. Analysis of variance was performed using STATISTICA software 10; significant differences were determined by the Duncan multiple range test to separate the means of the parameters measured.

Treatments	Plant Height	Stem Diameter	Number of Fruits	Total Fruit Weight	Total Root Weight
	cm	mm		Grams	Grams
Solution + BD163	41.25 ± 1.03 ab	9.04 ± 0.25 a	1.25 ± 0.25 a	25.75 ± 8.54 a	69.00 ± 17.78 a
Solution	44.00 ± 3.29 a	9.55 ± 0.62 a	1.50 ± 0.29 a	98.5 ± 44.81 a	34.00 ± 14.70 a
BD163	35.25 ± 3.86 b	9.71 ± 0.39 a	1.25 ± 0.25 a	25.25 ± 12.46 a	48.75 ± 19.59 a
Control (No Solution + No BD163)	41.75 ± 0.63 ab	9.22 ± 0.49 a	1.75 ± 0.25 a	96.75 ± 37.13 a	20.00 ± 3.14 a
*p* Value	0.16 ns	0.73 ns	0.50 ns	0.18 ns	0.18 ns

**Table 2:** Measurement of physiological/gas exchange parameters of tomato plants inoculated with *Pectobacterium carotovorum* strain BD163 (BD163) and treated with a nutrient solution containing CaCO_3_ (2 mM), NaCl (1 mM) and K_2_Cr_2_O_7_ (0.001 mM) (solution). Letters accompanying the measurements indicate statistically significant differences at (*p <* 0.05) *n* = 4. Analysis of variance was performed using STATISTICA software 10; significant differences were determined by the Duncan multiple range test to separate the means of the parameters measured. * The ratio of intercellular to ambient carbon dioxide concentration.

Treatments	Photosynthesis Rate	Stomatal Conductance	Intercellular CO_2_ Concentration	Transpiration Rate	Ci/Ca *	Water Use Efficiency
	µmol(CO_2_) m^−2^ s^−1^	mol(H_2_O) m^−2^ s^−1^	mol m^−2^ s^−1^	mol (H_2_O) ms^−2^ s^−1^	µmol (CO_2_) m^−2^ s^−1^	µmol (CO_2_) m^−1^ H_2_O
Solution + BD163	10.54 ± 1.39 a	0.09 ± 0.01 a	216.72 ± 26.19 a	2.98 ± 0.19 a	0.55 ± 0.06 a	114.73 ± 15.08 b
Solution	9.73 ± 1.32 a	0.08 ± 0.02 a	198.18 ± 22.99 a	2.65 ± 0.44 a	0.46 ± 0.05 a	128.35 ± 15.08 b
BD163	12.39 ± 1.00 a	0.07 ± 0.01 a	214.05 ± 14.55 a	2.09 ± 0.16 a	0.41 ± 0.06 a	208.79 ± 29.76 a
Control (No Solution + No BD163)	10.51 ± 1.56 a	0.09 ± 0.01 a	213.14 ± 10.18 a	2.75 ± 0.22 a	0.50 ± 0.02 a	116.11 ± 4.90 b
*p* Value	0.56 ns	0.47 ns	0.91 ns	0.19 ns	0.50 ns	0.01 s

**Table 3:** Measurement of elements (in parts per million) of tomato plants inoculated with *Pectobacterium carotovorum* strain BD163 (BD163) and treated with a nutrient solution containing CaCO_3_ (2 mM), NaCl (1 mM) and K_2_Cr_2_O_7_ (0.001 mM) (solution). Letters accompanying the measurements indicate statistically significant differences at (*p <* 0.05) *n* = 4. Analysis of variance was performed using STATISTICA software 10; significant differences were determined by the Duncan multiple range test to separate the means of the parameters measured.

Treatments	Aluminum	Boron	Bismuth	Calcium	Potassium	Nickel
Solution + BD163	0.06 ± 0.02 a	0.19 ± 0.01 a	0.47 ± 0.01 b	40.31 ± 4.56 a	39.76 ± 6.14 a	0.02 ± 0.00 a
Solution	0.06 ± 0.01 a	0.17 ± 0.01 ab	0.40 ± 0.01 b	33.58 ± 2.78 a	41.48 ± 1.95 a	0.01 ± 0.00 b
BD163	0.09 ± 0.01 a	0.14 ± 0.01 bc	0.35 ± 0.02 ab	38.93 ± 6.54 a	53.38 ± 7.63 a	0.01 ± 0.00 b
Control (No Solution + No BD163)	0.08 ± 0.02 a	0.10 ± 0.13 c	0.13 ± 0.16 a	25.05 ± 11.57 a	32.45 ± 11.98 a	0.01 ± 0.00 b
*p* Value	0.58 ns	0.05 s	0.06 s	0.45 ns	0.34 ns	0.04 s

**Table 4:** Pearson correlation coefficients of measurements of growth, yield, physiology/gas exchange and elements of tomato plants inoculated with *Pectobacterium carotovorum* (strain BD163) and treated with a nutrient solution containing CaCO_3_ (2 mM), NaCl (1 mM) and K_2_Cr_2_O_7_ (0.001 mM) (solution). Data for all four experimental treatments, “BD163 + solution”, “solution”, “BD163” and “control” (no Solution + no BD163), was pooled to generate the correlation table. The parameters measured were plant height (PH), stem diameter (SD), number of fruits (N/f), total fruit weight (TFW), total root weight (TRW), rate of photosynthesis (Photo), stomatal conductance (Stoma), intercellular CO_2_ (Inter), transpiration rate (Trans), the ratio of intercellular to ambient carbon dioxide concentration (Ci/Ca), water use efficiency (WUE), and the quantities of the elements aluminum (Al), boron (B), bismuth (Bi), calcium (Ca), potassium (K) and nickel (Ni).

	PH	SD	N/f	TFW	TRW	Photo	Stoma	Inter	Trans	Ci/Ca	WUE	Al	B	Bi	Ca	K	Ni
PH	1	−0.464	0.611	0.722	−0.302	−0.999	0.669	−0.573	0.784	0.711	−0.895	−0.859	0.209	−0.017	−0.437	−0.772	0.027
SD		1	−0.32	0.027	−0.256	0.464	−0.946	−0.453	−0.906	−0.932	0.804	0.508	−0.181	0.016	0.127	0.747	−0.623
N/f			1	0.866	−0.831	−0.571	0.592	−0.228	0.418	0.605	−0.626	−0.137	−0.638	−0.801	−0.971	−0.853	−0.528
TFW				1	−0.871	−0.693	0.298	−0.683	0.255	0.335	−0.51	−0.292	−0.431	−0.567	−0.851	−0.635	−0.667
TRW					1	0.259	−0.045	0.441	0.128	−0.062	0.139	−0.215	0.799	0.843	0.924	0.424	0.91
Photo						1	−0.659	0.578	−0.788	−0.702	0.891	0.883	−0.257	−0.033	0.392	0.749	−0.063
Stoma							1	0.22	0.942	0.998	−0.929	−0.567	0.019	−0.212	−0.405	−0.921	0.372
Inter								1	0.035	0.164	0.148	0.448	−0.153	−0.11	0.22	0.035	0.463
Trans									1	0.954	−0.962	−0.802	0.327	0.09	−0.193	−0.823	0.521
Ci/Ca										1	−0.949	−0.605	0.038	−0.197	−0.413	−0.93	0.357
WUE											1	0.784	−0.153	0.096	0.423	0.919	−0.27
Al												1	−0.675	−0.482	−0.08	0.489	−0.468
B													1	0.969	0.788	0.245	0.775
Bi														1	0.902	0.477	0.717
Ca															1	0.712	0.696
K																1	0.01
Ni																	1

## Data Availability

Data are contained within the article and Supplementary Materials.

## Supplementary Materials

The following supporting information can be downloaded at https://www.mdpi.com/article/10.3390/plants14121876/s1, Figure S1: An OPLS-DA S-plot utilizing Pareto scaling with mean centering to compare control and treated tomato plants. The UHPLC-qTOF-MS (Negative mode) data sets of tomato leaf samples compared to control. The treatments were, inoculation with *Pectobacterium carotovorum* (strain BD163) and treated with a nutrient solution containing CaCO_3_ (2 mM), NaCl (1 mM) and CR_2_K_2_0_7_ (0.001 mM) **(SolutionBD163)**; only the nutrient solution **(Solution)**; only *Pectobacterium* **(BD163)**. (**A**) (Control vs BD163) (**B**) (Control vs Solution) (**C**) (Control vs BD163+Solution); Table S1: The annotated metabolites in tomato plants treated as follows, inoculation with *Pectobacterium carotovorum* (strain BD163) and treated with a nutrient solution containing CaCO_3_ (2mM), NaCl (1mM) and CR_2_K_2_0_7_ (0.001mM) **(Solution + BD163)**; only the nutrient solution **(Solution)**; only *Pectobacterium* **(BD163)**, exhibit concentration differences that are statistically significant between untreated (Control) and treated plants; Figure S2: Interactive heatmap analysis profiles of annotated metabolites from tomato leaves treated with *Pectobacterium carotovorum* and a nutrient solution containing CaCO_3_ (2mM), NaCl (1mM) and K_2_ Cr_2_0_7_ (0.001mM). The treatments were, inoculation with *P. carotovorum* (strain BD163) and treated with a nutrient solution **(SolutionBD163)**; only the nutrient solution **(Solution)**; only *Pectobacterium* **(BD163)**. (**A**) (Control vs BD163) (**B**) (Control vs Solution) (**C**) (Control vs BD163Solution); Figure S3: Computed orthogonal partial least squares—discriminant analysis (OPLS-DA) showing separated metabolic features of tomato leaves with two treatments, namely, tomato inoculated with *Pectobacterium carotovorum* strain BD163 (BD163—Red circle) and the untreated control (Control—green circle). Analysis with the LCMS-9030 qTOF was done on leaves sampled 27 days after exposure to the treatments; Figure S4: Computed orthogonal partial least squares—discriminant analysis (OPLS-DA) showing separated metabolic features of tomato leaves with four treatments, namely, tomato inoculated with *Pectobacterium carotovorum* (strain BD163) and treated with a nutrient solution containing CaCO_3_ (2mM), NaCl (1mM) and K_2_Cr_2_O_7_ (0.001mM) (Solution + BD163—green circle); and the untreated control (Control—purple circle). Analysis with the LCMS-9030 qTOF was done on leaves sampled 27 days after exposure to the treatments.
