# Supplementary material for: Physiological, Chemical and Metabolite Profiling of Pectobacterium carotovorum-Inoculated Tomato Plants Grown in Nutrient-Amended Soils
Source: Plants (Basel). 2025 Jun 18;14(12):1876. doi: 10.3390/plants14121876 (PMC12196568; doi:10.3390/plants14121876)
Supplement: Supplementary file 1 [file plants-14-01876-s001.zip › plants-3559401-supplementary.pdf]

Physiological, chemical and metabolite profiling of *Pectobacterium carotovorum*-inoculated tomato plants grown in nutrient amended soils

[1]Sandra Maluleke, [1]Udoka Vitus Ogugua, [2]Njabulo Mdluli, [3]Ntakadzeni Edwin Madala and [1*]Khayalethu Ntushelo

Supplementary figures and table

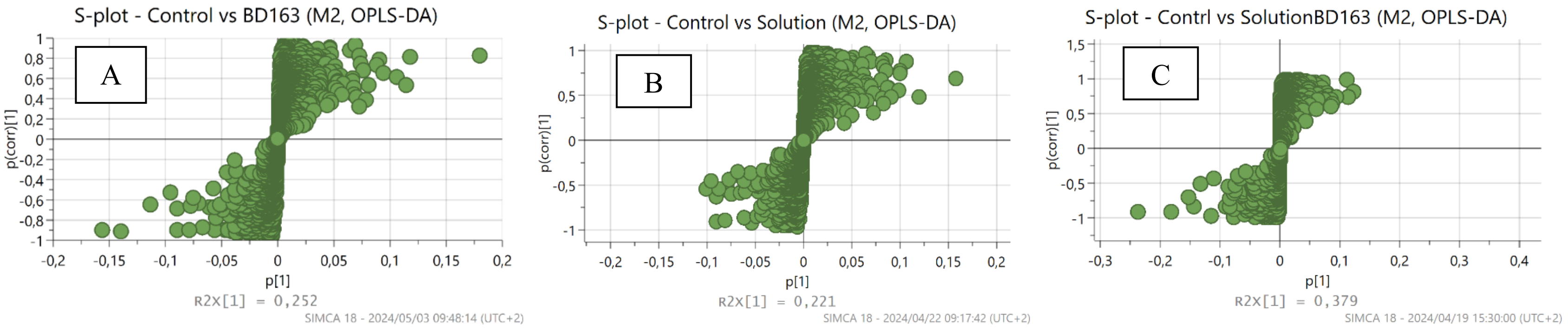

Supplementary Figure S1: An OPLS-DA S-plot utilizing Pareto scaling with mean centering to compare control and treated tomato plants. The UHPLC-qTOF-MS (Negative mode) data sets of tomato leaf samples compared to control. The treatments were, inoculation with *Pectobacterium carotovorum* (strain BD163) and treated with a nutrient solution containing $CaCO_3$ (2mM), NaCl (1mM) and $CR_2K_20_7$ (0.001mM) **(SolutionBD163)**; only the nutrient solution **(Solution)**; only *Pectobacterium* **(BD163)**. **A** (Control vs BD163) **B** (Control vs Solution) **C** (Control vs BD163+Solution)

Supplementary Table S1: The annotated metabolites in tomato plants treated as follows, inoculation with *Pectobacterium carotovorum* (strain BD163) and treated with a nutrient solution containing $CaCO_3$ (2mM), NaCl (1mM) and $CR_2K_20_7$ (0.001mM) **(Solution + BD163)**; only the nutrient solution **(Solution)**; only *Pectobacterium* **(BD163),** exhibit concentration differences that are statistically significant between untreated (Control) and treated plants.

**Molecular Feature**	**Molecular formula**	**Compound and category**	**m/z**	**Rt (min)**	**Untreated**	**Treated**	**log2fold**	**VIP score**	**Adducts**
**BD163**									
M609T5_4	$C_{27}H_{30}O_{16}$	Phytomelin	609,158	4,84	18,09	20,25	-2,16	3,23	$[M-H]^-$
M611T5_20	$C_{38}H_{44}O_7$	Gambogic aldehyde	611,151	4,64	18,81	19,72	-0,91	2,32	$[M-H]^-$
M759T12	$C_{41}H_{77}NO_{11}$	tetradecy /clohexyl-4-hydr 2-hydroxy-3-(E)-3-hydroxy-1-(2R,3R,45,5R,6R)-3,4,5-trihydroxy-6-(hydroxymethyl) oxan-2-yl] 3R,45,55)-3-(h oxyoctadec-4-en-2-yljamino]-3-oxopropanoate	758,541	12,13	18,39	18,99	-0,61	4,15	
M391T1_4	$C_{11}H_{15}N_4O_7P$	Polydeazaadenylic acid	391,068	0,73	14,61	17,61	-3,00	2,76	$[M+CH_2O_2-H]^-$
M457T11_3	$C_{30}H_{50}O_3^-$	4-butoxy-3-[(E)-7-hydroxy-7,11-dimethylhexadec-2-enyl]-5,6-dimethylcyclohex-2-en-1-ol	457,371	10,91	18,95	19,99	-1,04	5,78	$[M-H]^-$
M499T5_1	$C_{22}H_{30}O_{14}$	6-methoxy-7-[(2S,3R,4S,5S,6R)-3,4,5-trihydroxy-6-[[(2R,3R,4R,5R,6S)-3,4,5-trihydroxy-6-methyloxan-2-yl] oxymethyl] oxan-2-yl] oxychromen-2-one	499,144	5,34	18,33	19,15	-0,82	3,97	$[M+H_2O-H]^-$
M787T6_2	$C_{39}H_{64}O_{16}$	Atroviolaceoside	787,411	5,96	17,99	18,85	-0,86	2,42	$[M-H]^-$
M593T5_1	$C_{27}H_{30}O_{15}$	Nicotifolin	593,150	5,12	20,00	20,90	-0,90	5,40	$[M-H]^-$

M249T5_2	$C_{14}H_{10}N_4O$	6-Phenyl-2-(pyrazin-2-yl) pyrimidin-4-ol	249,079	5,31	18,58	19,15	-0,57	2,83	$[M-H]^-$
M594T5_18	$C_{20}H_{26}N_{11}O_9P$	[4-Amino-5-(6-aminopurin-9-yl)-3-hydroxyoxolan-2-yl] methyl [5-(6-aminopurin-9-yl)-4-hydroxy-2-methoxyoxolan-3-yl] hydrogen phosphate	594,153	5,12	18,31	19,38	-1,08	4,17	$[M-H]^-$
M555T10	$C_{24}H_{46}O_9S$	1,18-Bis(2-methoxyethoxy)-1,18-dioxooctadecane-9-sulfonic acid	555,283	9,53	21,44	20,02	1,42	12,12	$[M-H]^-$
M329T7_2	$C_{18}H_{34}O_5$	(Z)-9,12,13-trihydroxyoctadec-10-enoic acid	329,232	6,58	20,85	20,08	0,77	2,43	$[M-H]^-$
M271T9	$C_{16}H_{32}O_3$	2-Hydroxyhexadecanoic acid	271,227	8,91	18,79	17,33	1,46	1,88	$[M-H]^-$
M556T10_2	$C_{22}H_{45}N_3O_8S$	*N*-[4-[2-[2-[2-[(2-hydroxy-2-methoxyethyl) amino]-2-oxoethoxy] ethoxy] ethylamino]butylsulfonyl]nonanamide	556,287	9,72	19,36	17,51	1,85	7,61	$[M+CH_2O_2-H]^-$
M611T5_21	$C_{27}H_{46}N_6O_{11}$	(4*S*)-4-[[(2*S*,3*S*)-2-[[(2*S*)-2-[[(2*S*)-2-[(2-aminoacetyl) amino]-3-carboxypropanoyl] amino]-3-methylbutanoyl] amino]-3-methylpentanoyl] amino]-5-[[(1*S*)-1-carboxy-2-methylpropyl] amino]-5-oxopentanoic acid	611,219	4,86	18,43	14,79	3,65	4,94	$[M+H_2O-H]^-$
M431T4_1	$C_{19}H_{30}O_8$	(4R)-4-hydroxy-3,5,5-trimethyl-4-[(E)-3-[(2S,3S,4R,5R,6S)-3,4,5-trihydroxy-6-(hydroxymethyl) oxan-2-yl] oxybut-1-enyl] cyclohex-2-en-1-one	431,191	4,34	20,58	19,87	0,71	9,43	$[M+CH_2O_2-H]^-$
M295T9_1	$C_{18}H_{32}O_3$	(10*E*)-9-hydroxyoctadeca-10,12-dienoic acid	295,227	8,75	18,31	17,19	1,12	3,87	$[M-H]^-$
M293T8_19	$C_{18}H_{32}O_4$	(10E,12Z) -(9S)-9-Hydroperoxyoctadeca-10,12-dienoic acid	293,211	8,45	19,46	18,34	1,13	5,63	$[M-H]^-$
M571T10_1	$C_{25}H_{49}O_{12}P$	1-Hexadecanoyl-sn-glycero-3-phospho-(1'-myo-inositol)	571,288	9,85	20,14	18,32	1,81	9,22	$[M-H]^-$
M307T7_2	$C_{18}H_{30}O_5$	2,3-dinor Prostaglandin E1	307,190	6,79	19,03	18,35	0,68	4,26	$[M+H_2O-H]^-$
M293T8_16	$C_{18}H_{30}O_3$	9-Hydroxy-10,12,15-octadecatrienoic acid	293,211	7,81	21,28	21,15	0,13	4,13	$[M-H]^-$

M407T10_2	$C_{19}H_{37}O_7P$	1-palmitoleoyl-*sn*-glycerol 3-phosphate	407,219	9,66	18,88	17,67	1,21	3,85	$[M - H]^{-}$
M191T1_7	$C_7H_{12}O_6$	Quinic acid	191,146	0,62	18,50	13,66	4,85	6,05	$[M - H]^{-}$
M431T9_2	$C_{21}H_{39}O_8P$	2-hydroxy-3-phosphonooxypropyl) (Z)-11-(3-pentyloxiran-2-yl) undec-9-enoate	431,219	8,96	18,75	17,74	1,01	3,96	$[M - H]^{-}$
M330T7_32	$C_{11}H_{27}N_9$	3-[2-(3-amino-3-iminopropyl) hydrazinyl]-2-[carbamimidoyl(ethyl)amino]-N',2-dimethylpropanimidamide	330,190	6,52	18,13	12,41	5,72	5,73	$[M + CH_2O_2 - H]^{-}$
M409T10_2	$C_{19}H_{39}O_7P$	1-Palmitoylglycerol 3-phosphate	409,235	10,01	20,39	19,87	0,52	7,00	$[M - H]^{-}$
M433T9_2	$C_{21}H_{39}O_7P$	[2-hydroxy-3-(octadeca-9,12-dienoyloxy) propoxy] phosphonic acid	433,235	9,41	20,24	19,54	0,70	6,05	$[M - H]^{-}$
M407T9_3	$C_{19}H_{37}O_7P$	1-palmitoleoyl-sn-glycerol 3-phosphate (2−)	407,219	9,44	19,67	18,90	0,77	3,38	
M577T9_1	$C_{27}H_{46}O_{11}S$	[(2S,3S,4S,5R,6S)-6-(2,3-dihydroxypropoxy)-3,5-dihydroxy-4-[(9Z,12Z,15Z)-octadeca-9,12,15-trienoyl] oxyoxan-2-yl]methanesulfonic acid	577,267	8,72	20,61	19,74	0,87	8,61	$[M - H]^{-}$
M293T8_15	$C_{18}H_{29}O_3$	9(R)-Hydroxy-10(E),12(Z),15(Z)-octadecatrienoic acid	293,175	7,81	20,89	20,19	0,71	5,46	$[M - H]^{-}$
**Solution**									
M459T11_1	$C_{25}H_{50}O_4$	17,25-Dihydroxypentacosanoic acid	459,373	11,50	18,75	19,50	-0,75	3,91	$[M + CH_2O_2 - H]^{-}$
M457T10_2	$C_{30}H_{52}O_4$	Protopanaxatriol	457,370	9,60	18,87	19,31	-0,44	2,73	[M + H2O− H] −
M191T1_2	$C_7H_{12}O_6$	Quinic acid	191,055	0,62	19,25	19,99	-0,74	4,39	$[M - H]^{-}$

M594T5_22	$C_{20}H_{26}N_{11}O_9P$	[4-Amino-5-(6-aminopurin-9-yl)-3-hydroxyoxolan-2-yl]methyl [5-(6-aminopurin-9-yl)-4-hydroxy-2-methoxyoxolan-3-yl] hydrogen phosphate	594,153	5,12	18,31	19,25	-0,94	2,53	$[M-H]^-$
M593T5_2	$C_{27}H_{30}O_{15}$	Kaempferol-3-O-rutinoside	593,150	5,12	20,00	20,79	-0,79	4,17	$[M-H]^-$
M457T11_2	$C_{30}H_{52}O_4$	(7R)-7-[(8R,9S,10S,13R,14S,17R)-3,7-dihydroxy-10,13-dimethyl-2,3,4,5,6,7,8,9,11,12,14,15,16,17-tetradecahydro-1H-cyclopenta[a]phenanthren-17-yl]-4-ethyl-3-methyloctanoic acid	457,371	11,50	22,40	22,54	-0,14	9,62	$[M+H_2O-H]^-$
M330T7_26	$C_{11}H_{27}N_9$	3-[2-(3-amino-3-iminopropyl) hydrazinyl]-2-[carbamimidoyl(ethyl)amino]-N',2-dimethylpropanimidamide	330,170	6,52	13,25	17,73	-4,48	5,96	$[M+H_2O-H]^-$
M191T1_1	$C_6H_8O_7$	Citric acid	191,019	0,72	20,71	20,97	-0,25	5,86	$[M-H]^-$
M610T5_21	$C_{21}H_{25}N_{11}O_9$	Adenosine carboxamido-adenosine	610,149	4,86	20,74	21,13	-0,39	4,08	$[M-H]^-$
M741T5_3	$C_{32}H_{38}O_{20}$	Quercetin 3-(2Gal-apiosylrobinobioside)	741,187	4,67	19,39	19,97	-0,57	4,98	$[M-H]^-$
M609T5_2	$C_{27}H_{30}O_{16}$	Phytomelin	609,145	4,89	21,86	22,05	-0,19	4,06	$[M-H]^-$
M275T1_2	$C_{12}H_6O_5$	4-Oxo-4H-(1) benzofuro(3,2-b)pyran-2-carboxylic acid	275,020	0,67	17,92	15,77	2,14	1,27	$[M+CH_2O_2-H]^-$
M295T8_25	$C_{18}H_{32}O_3$	(9S)-Hydroxy octadecadienoic acid	295,227	8,08	20,81	20,52	0,30	2,43	$[M-H]^-$
M556T10_1	$C_{22}H_{45}N_3O_8S$	*N*-[4-[2-[2-[2-[(2-hydroxy-2-methoxyethyl) amino]-2-oxoethoxy] ethoxy] ethylamino]butylsulfonyl]nonanamide	556,286	9,72	19,71	18,41	1,30	5,29	$[M+CH_2O_2-H]^-$
M311T8_2	$C_{18}H_{34}O_5$	6,7,10-Trihydroxy-8-octadecenoic acid	311,221	7,52	19,97	19,32	0,66	3,61	[M + H2O– H] –
M325T10	$C_{18}H_{30}O_3S$	4-Dodecylbenzene-1-(~2~H) sulfonic acid	325,183	9,69	21,36	21,04	0,32	6,49	$[M-H]^-$
M293T8_16	$C_{18}H_{30}O_3$	9-Hydroxy-10,12,15-octadecatrienoic acid	293,211	7,80	21,28	21,05	0,23	3,14	$[M-H]^-$

M291T8_2	$C_{18}H_{28}O_3$	9-Oxo-10,12,15-octadecatrienoic acid	291,195	7,87	20,21	19,90	0,30	3,69	$[M-H]^-$
M611T5_21	$C_{27}H_{46}N_6O_{11}$	(4*S*)-4-[[(2*S*,3*S*)-2-[[(2*S*)-2-[[(2*S*)-2-[(2-aminoacetyl) amino]-3-carboxypropanoyl] amino]-3-methylbutanoyl] amino]-3-methylpentanoyl] amino]-5-[[(1*S*)-1-carboxy-2-methylpropyl] amino]-5-oxopentanoic acid	611,220	4,86	18,43	16,38	2,06	4,63	$[M+H_2O-H]^-$
M271T9	$C_{16}H_{32}O_3$	2-Hydroxyhexadecanoic acid	271,227	8,91	18,79	17,96	0,83	2,03	$[M-H]^-$
M311T9_2	$C_{17}H_{28}O_3S$	4-undecylbenzenesulfonic acid	311,167	9,15	21,12	20,74	0,38	6,99	[M – H] –
M409T10_2	$C_{19}H_{39}O_7P$	1-Palmitoylglycerol 3-phosphate	409,235	10,01	20,39	19,71	0,68	7,77	[M – H] –
M572T10	$C_{26}H_{41}N_9O_7$	(2S)-2-[[4-[(2-amino-4-oxo-2,3,4a,5,6,7,8,8a-octahydro-1H-pteridin-6-yl) methylamino] benzoyl] amino]-5-[[7-(hydroxyamino)-7-oxoheptyl]amino]-5-oxopentanoic acid	572,291	9,85	18,31	16,97	1,34	4,94	[M – H] –
M571T10_1	$C_{25}H_{49}O_{12}P$	1-Hexadecanoyl-sn-glycero-3-phospho-(1'-myo-inositol)	571,288	9,85	20,14	18,79	1,35	9,34	$[M-H]^-$
M407T10_2	$C_{19}H_{37}O_7P$	1-palmitoleoyl-*sn*-glycerol 3-phosphate	407,219	9,66	18,88	17,79	1,09	3,73	$[M-H]^-$
M309T7_1	$C_{18}H_{30}O_4$	(9R,10E,12Z,15Z)-9-Hydroperoxyoctadeca-10,12,15-trienoate	309,206	7,24	20,53	20,10	0,44	4,22	$[M-H]^-$
M555T10_2	$C_{24}H_{46}O_9S$	1,18-Bis(2-methoxyethoxy)-1,18-dioxooctadecane-9-sulfonic acid	555,283	9,52	20,68	19,95	0,73	7,07	$[M-H]^-$
**Solution + BD163.**									
M594T5_21	$C_{20}H_{26}N_{11}O_9P$	[4-Amino-5-(6-aminopurin-9-yl)-3-hydroxyoxolan-2-yl] methyl [5-(6-aminopurin-9-yl)-4-hydroxy-2-methoxyoxolan-3-yl] hydrogen phosphate	594,153	5,12	18,31	19,38	-1,07	3,03	$[M-H]^-$

M457T11_2	$C_{30}H_{52}O_4$	(7R)-7-[(8R,9S,10S,13R,14S,17R)-3,7-dihydroxy-10,13-dimethyl-2,3,4,5,6,7,8,9,11,12,14,15,16,17-tetradecahydro-1H-cyclopenta[a]phenanthren-17-yl]-4-ethyl-3-methyloctanoic acid	457,371	11,50	22,40	23,50	-1,11	8,13	[M + H_2O− H]⁻
M459T11_2	$C_{25}H_{50}O_4$	17,25-Dihydroxypentacosanoic acid	459,373	11,50	18,75	20,52	-1,78	3,99	[M + CH_2O_2− H]⁻
M385T12_1	$C_{26}H_{42}O_2$	(8Z,11Z,14Z,17Z,20Z)-Hexacosapentaenoic acid	385,313	11,50	17,56	19,18	-1,63	2,48	[M − H]⁻
M610T5_22	$C_{21}H_{25}N_{11}O_9$	Adenosine carboxamido-adenosine	610,149	4,86	20,74	21,19	-0,45	5,49	[M − CI]⁻
M325T10_2	$C_{18}H_{30}O_3S$	4-Dodecylbenzene-1-(~2~H) sulfonic acid	325,183	9,69	21,36	21,76	-0,40	5,86	[M − H]⁻
M191T1_2	$C_7H_{12}O_6$	Quinic acid	191,055	0,62	19,25	20,19	-0,94	3,83	[M − H]⁻
M717T12	$C_{39}H_{76}NO_8P$	[(2R)-1-[2-aminoethoxy(hydroxy)phosphoryl] oxy-3-decanoyloxypropan-2-yl] (Z)-tetracos-15-enoate	716,522	11,89	4,91	19,68	-14,77	3,50	[M − H]⁻
M489T10_3	$C_{27}H_{54}O_5S$	1-Methoxy-1-oxohexacosane-2-sulfonic acid	489,360	9,68	17,19	19,62	-2,43	6,06	[M − H]⁻
M457T10_1	$C_{30}H_{52}O_4$	Protopanaxatriol	457,371	9,60	19,85	22,08	-2,22	12,77	[M + H_2O− H]⁻
M717T9_1	$C_{39}H_{76}NO_8P$	[1-[2-aminoethoxy(hydroxy)phosphoryl] oxy-3-dodecanoyloxypropan-2-yl] (Z)-docos-13-enoate	716,522	9,24	8,65	19,26	-10,61	6,24	[M − H]⁻
M609T5_3	$C_{27}H_{30}O_{16}$	Phytomelin	609,145	4,84	21,86	22,08	-0,22	6,99	[M − H]⁻
M535T9_1	$C_{28}H_{58}O_2S_2$	1-Tetradecylsulfonylsulfanyltetradecane	535,384	9,20	16,93	20,05	-3,12	6,27	[M + CH_2O_2− H]⁻
M457T9_3	$C_{30}H_{52}O_4$	(3R,5R,8R,9R,10R,12R,13R,14R,17S)-17-[(E,2S)-2,6-dihydroxy-6-methylhept-4-en-2-yl]-4,4,8,10,14-pentamethyl-2,3,5,6,7,9,11,12,13,15,16,17-dodecahydro-1H-cyclopenta[a]phenanthrene-3,12-diol	457,371	9,21	19,85	22,26	-2,40	20,07	[M + H_2O− H]⁻

M458T9_1	$C_{28}H_{49}N_3O_2$	N-[3-[[2-(hexadecyl amino) acetyl] amino]-2-methylphenyl] propenamide	458,374	9,20	18,37	20,41	-2,04	17,76	$[M - H]^-$
M291T8_2	$C_{18}H_{28}O_3$	9-Oxo-10,12,15-octadecatrienoic acid	291,195	7,87	20,21	19,64	0,57	1,55	$[M - H]^-$
M295T8_25	$C_{18}H_{32}O_3$	(9S)-Hydroxy octadecadienoic acid	295,227	8,09	20,81	20,38	0,43	1,54	$[M - H]^-$
M293T8_14	$C_{18}H_{30}O_3$	9-Hydroxy-10,12,15-octadecatrienoic acid	293,211	7,80	21,28	20,91	0,38	2,58	$[M - H]^-$
M311T8_2	$C_{18}H_{34}O_5$	6,7,10-Trihydroxy-8-octadecenoic acid	311,221	7,52	19,97	19,28	0,70	2,41	$[M + H_2O - H]^-$
M309T7_1	$C_{18}H_{30}O_4$	(9R,10E,12Z,15Z)-9-Hydroperoxyoctadeca-10,12,15-trienoate	309,206	7,24	20,53	19,48	1,06	4,52	$[M - H]^-$
M409T10_2	$C_{19}H_{39}O_7P$	1-Palmitoylglycerol 3-phosphate	409,235	10,01	20,39	19,27	1,12	4,49	$[M - H]^-$
M433T9_2	$C_{21}H_{39}O_7P$	1-Linoleoyl-3-glycerophosphate	433,235	9,41	20,24	18,35	1,90	5,43	$[M - H]^-$
M431T4_1	$C_{19}H_{30}O_8$	Roseoside	431,191	4,33	20,58	20,01	0,57	4,12	$[M + CH_2O_2 - H]^-$
M555T10_1	$C_{24}H_{46}O_9S$	1,18-Bis(2-methoxyethoxy)-1,18-dioxooctadecane-9-sulfonic acid	555,283	9,52	20,69	18,72	1,98	6,75	$[M + CH_2O_2 - H]^-$
M293T8_16	$C_{18}H_{32}O_4$	(10E,12Z) -(9S)-9-Hydroperoxyoctadeca-10,12-dienoic acid	293,211	8,45	19,46	18,25	1,21	4,12	$[M + H_2O - H]^-$
M556T10_1	$C_{22}H_{41}N_9O_9$	H-Arg-Ala-Asp-Ser-Lys-OH	556,287	9,52	18,91	16,99	1,92	3,68	$[M + H_2O - H]^-$
M571T10_1	$C_{25}H_{48}O_{12}P^-$	1-Palmitoylglycerophosphoinositol	571,288	9,85	20,14	17,54	2,59	6,04	[M − H] −

M577T9_1	$C_{27}H_{46}O_{11}S$	[(2S,3S,4S,5R,6S)-6-(2,3-dihydroxypropoxy)-3,5-dihydroxy-4-[(9Z,12Z,15Z)-octadeca-9,12,15-trienoyl] oxyoxan-2-yl]methanesulfonic acid	577,267	8,72	20,61	18,38	2,23	8,17	[M − H] −

Analysed using high-definition MS in UHPLC negative mode, this summary presents the annotated metabolites found in tomato leaves from BD163, solution and BD163Solution treated leaf respectively. The metabolites that played a role in the distinguishing differences in the changed metabolomes were determined using OPLS-DA S plots, with a threshold score values 0.05. Annotations are associated with the detection of metabolites using MS2. The concentrations of metabolites are scaled using a logarithmic base of 2. The metabolites shown are the ones that exhibit differences between the treated and untreated samples.

Heat Map Correlation Control vs BD163.

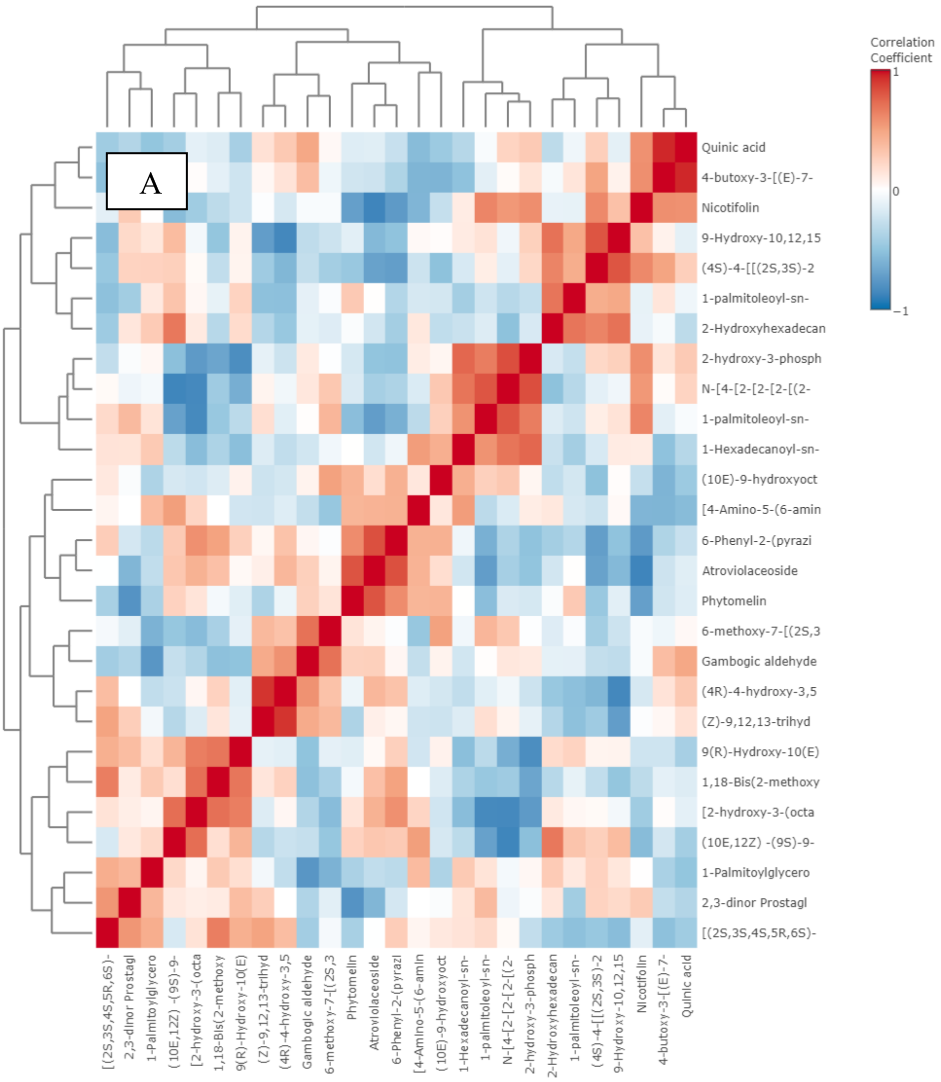

Heat Map Correlation Control vs Solution.

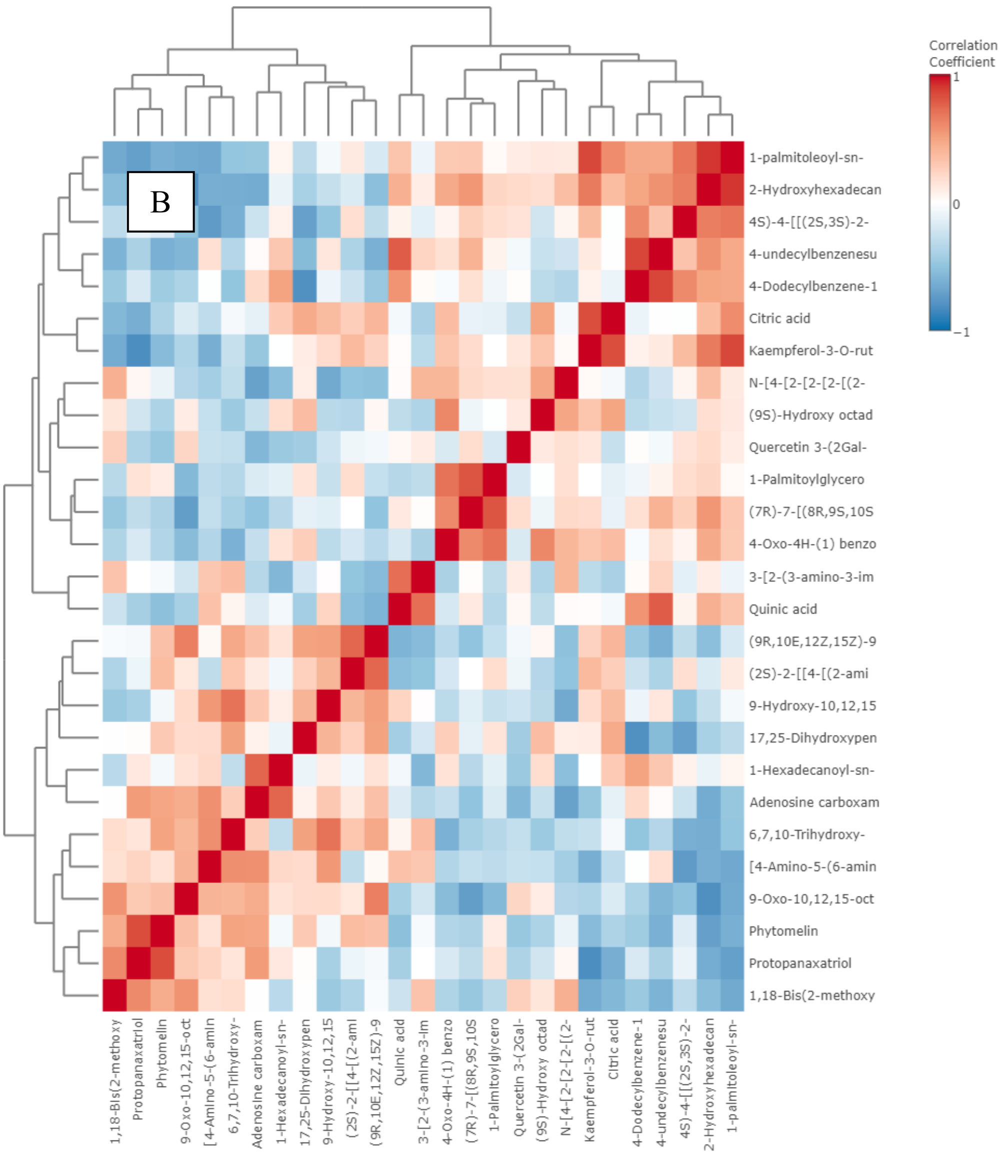

Heat Map Correlation Control vs BD163Solution.

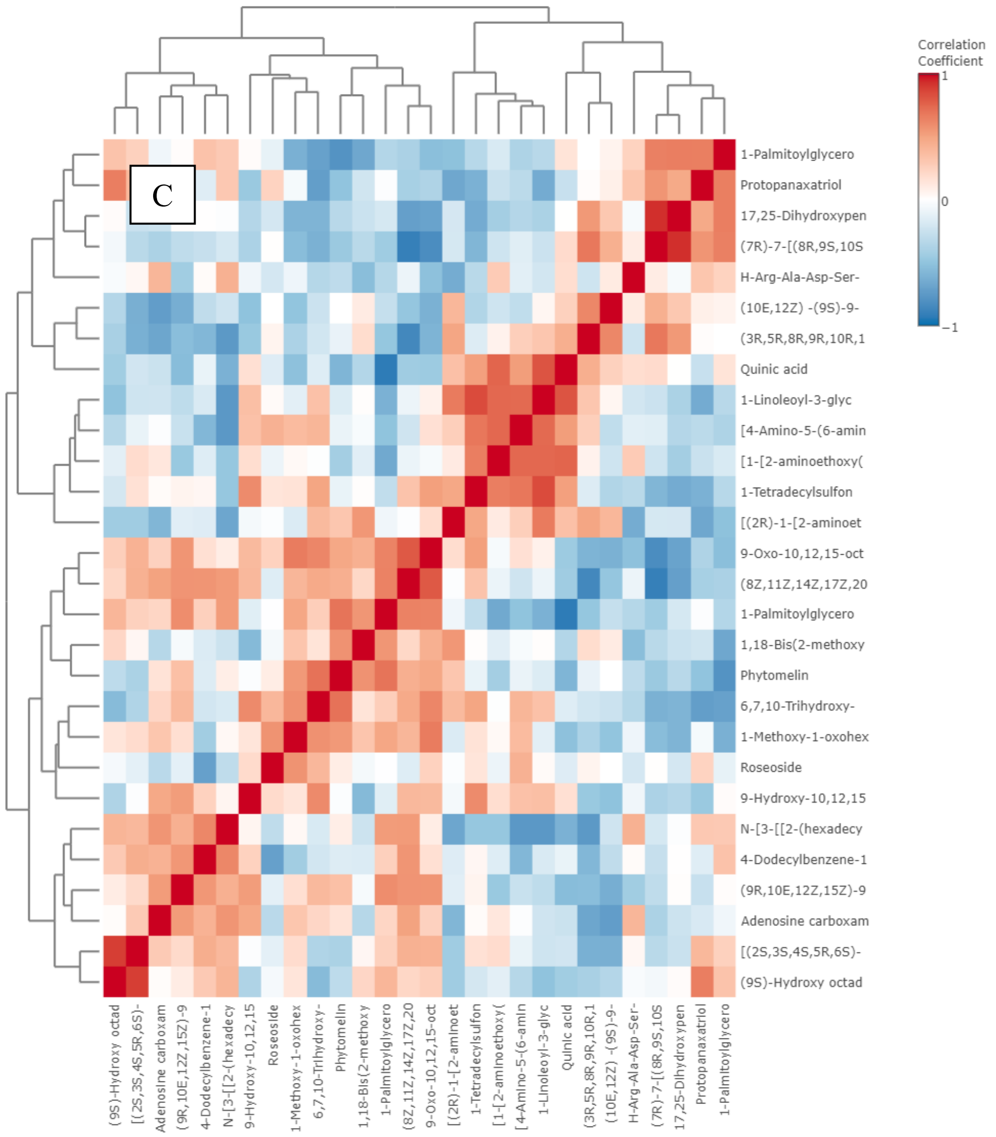

Supplementary Figure S2. Interactive heatmap analysis profiles of annotated metabolites from tomato leaves treated with *Pectobacterium carotovorum* and a nutrient solution containing $CaCO_3$ (2mM), NaCl (1mM) and K_2 Cr_20_7 (0.001mM). The treatments were, inoculation with *P. carotovorum* (strain BD163) and treated with a nutrient solution **(SolutionBD163)**; only the nutrient solution **(Solution)**; only *Pectobacterium* **(BD163)**. **A** (Control vs BD163) **B** (Control vs Solution) **C** (Control vs BD163Solution).

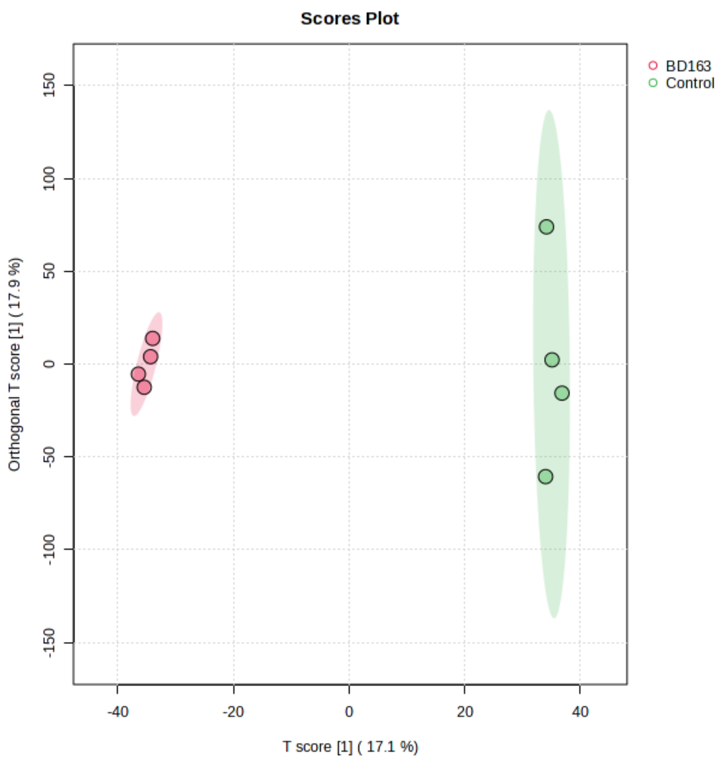

Supplementary Figure S3. Computed orthogonal partial least squares – discriminant analysis (OPLS-DA) showing separated metabolic features of tomato leaves with two treatments, namely, tomato inoculated with *Pectobacterium carotovorum* strain BD163 (BD163 – Red circle) and the untreated control (Control – green circle). Analysis with the LCMS-9030 qTOF was done on leaves sampled 27 days after exposure to the treatments.

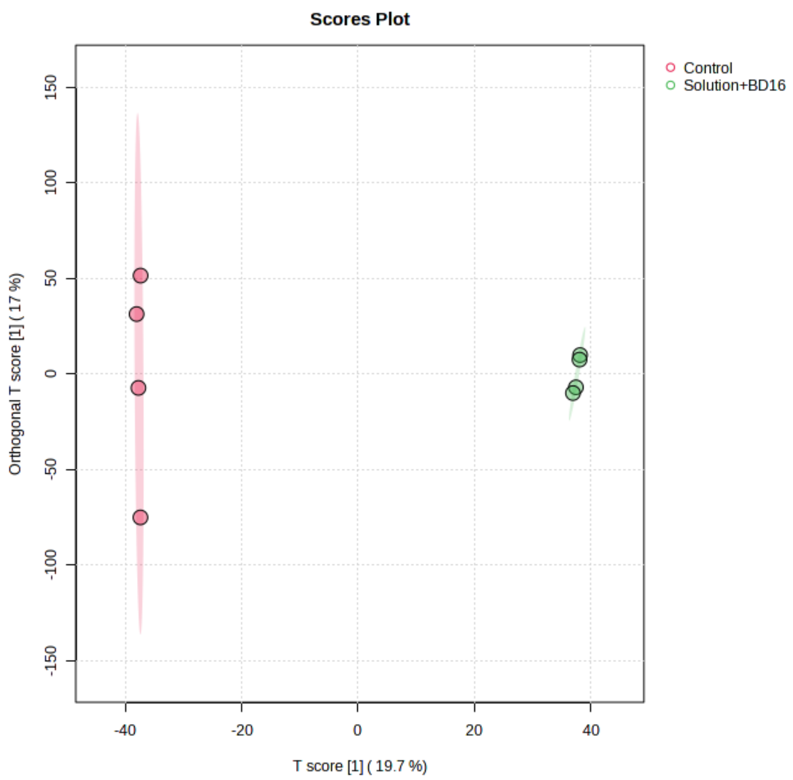

Supplementary Figure S4. Computed orthogonal partial least squares – discriminant analysis (OPLS-DA) showing separated metabolic features of tomato leaves with four treatments, namely, tomato inoculated with *Pectobacterium carotovorum* (strain BD163) and treated with a nutrient solution containing $CaCO_3$ (2mM), NaCl (1mM) and $K_2Cr_2O_7$ (0.001mM) (Solution + BD163 – green circle); and the untreated control (Control – purple circle). Analysis with the LCMS-9030 qTOF was done on leaves sampled 27 days after exposure to the treatments.

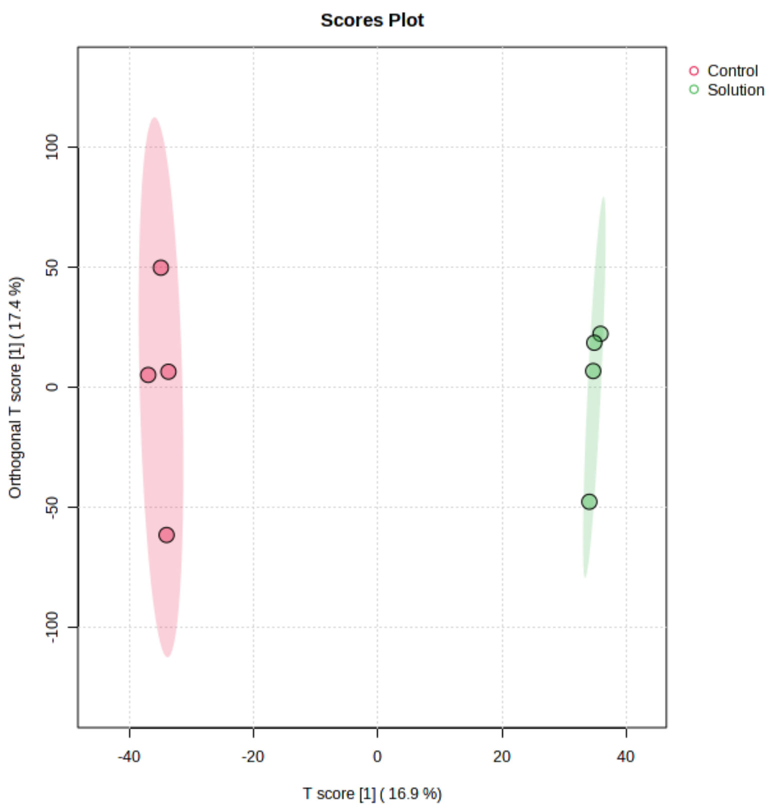

Supplementary Figure S5. Computed orthogonal partial least squares – discriminant analysis (OPLS-DA) showing separated metabolic features of tomato leaves with four treatments, namely, tomato treated with a nutrient solution containing $CaCO_3$ (2mM), NaCl (1mM) and $K_2Cr_2O_7$ (0.001mM) (Solution– green circle); the untreated control (Control – red circle). Analysis with the LCMS-9030 qTOF was done on leaves sampled 27 days after exposure to the treatments.
